# Supplementary material for: Chip-based multimodal super-resolution microscopy for histological investigations of cryopreserved tissue sections
Source: Light Sci Appl. 2022 Feb 24;11:43. doi: 10.1038/s41377-022-00731-w (PMC8873254; doi:10.1038/s41377-022-00731-w)
Supplement: Supplementary file 1 — Supplementary Information [file 41377_2022_731_MOESM1_ESM.docx]

***Supplementary information***

Chip-based multimodal super-resolution microscopy for histological investigations of cryopreserved tissue sections

Luis E. Villegas-Hernández^1,#^, Vishesh Dubey^1,#^, Mona Nystad^2,3^, Jean-Claude Tinguely^1^, David A. Coucheron^1^, Firehun T. Dullo^1^, Anish Priyadarshi^1^, Sebastian Acuña^1^, Azeem Ahmad^1^, José M. Mateos^4^, Gery Barmettler^4^, Urs Ziegler^4^, Åsa Birna Birgisdottir^5,6^, Aud-Malin Karlsson Hovd^7^, Kristin Andreassen Fenton^7^, Ganesh Acharya^2,8^, Krishna Agarwal^1^, Balpreet Singh Ahluwalia^1,8,*^

^1^ Department of Physics and Technology, UiT The Arctic University of Norway, Klokkargårdsbakken N-9019, Tromsø, Norway

^2^ Department of Clinical Medicine, Women’s Health and Perinatology Research Group, UiT The Arctic University of Norway, Tromsø, Norway

^3^ Department of Obstetrics and Gynecology, University Hospital of North Norway, Tromsø, Norway

^4^ Center for Microscopy and Image Analysis, University of Zurich, Zürich, Switzerland

^5^ Division of Cardiothoracic and Respiratory Medicine, University Hospital of North Norway, Tromsø, Norway

^6^ Department of Clinical Medicine, Clinical Cardiovascular Research Group, UiT The Arctic University of Norway, Tromsø, Norway

^7^ Department of Medical Biology, RNA and Molecular Pathology Research Group, UiT The Arctic University of Norway, Tromsø, Norway

^8^ Division of Obstetrics and Gynecology, Department of Clinical Science, Intervention and Technology, Karolinska Institute, Stockholm, Sweden

^#^ These authors contributed equally to this work

^*^ Corresponding author: balpreet.singh.ahluwalia@uit.no

| **#** | **Title** | **Supplementary page** |
| --- | --- | --- |

S1. Evanescent field simulations II

S2. Sample preparation work-flow for chip-TIRFM of Tokuyasu sections III

S3. Mode-averaging for homogeneous illumination in chip-TIRFM imaging IV

S4. Photobleaching of membrane markers V

S5. Sectioning artifacts VI

S6. Materials and reagents used for the preparation of Tokuyasu sections VII

S7. Chip-TIRFM imaging of immunolabeled mouse kidney samples VIII

S8. Chip-TIRFM imaging of immunolabeled human kidney samples IX

S9. Chip-TIRFM imaging of Pig heart tissue X

S10. Large FOV imaging of paraffin-embedded samples using chip-TIRFM XI

S11. Quantification of resolution improvement based on decorrelation analysis XII

S12. Comparative FOV between chip-based IFON and SIM XIII

S13. Detailed description of the chip-TIRFM setup XIV

S14. SEM imaging on a photonic chip XV

S15. References – Supplementary Information XVI

1. Evanescent field simulations

For the estimation of the waveguide parameters such as surface intensity and extent of evanescent field, simulations with Fimmwave (Photon Design) were performed for a strip waveguide having a width of 200 µm. Figure S1a shows the schematic diagram of a strip waveguide. The waveguides are fabricated on the SiO_2_ coated Si substrate. Figure S1b shows the distribution of the fundamental TE mode along the width and core thickness of the waveguide structure. The coupled light propagates through the length of the waveguide generating an evanescent field on its top. The surface intensity of the evanescent field depends highly on the geometry of the waveguide, refractive index differences of the core and surrounding material, and the wavelength of the coupled light. Figure S1c shows the variation in surface intensity and relative depth of the evanescent field (penetration depth) as a function of core thickness. As the core thickness increases, the surface intensity decreases dramatically. The amplitude of the penetration depth also decreases with increasing core thickness and becomes almost stable after 150 nm. The simulation results allow choosing a core thickness between 150 nm and 250 nm for chip-TIRFM applications.


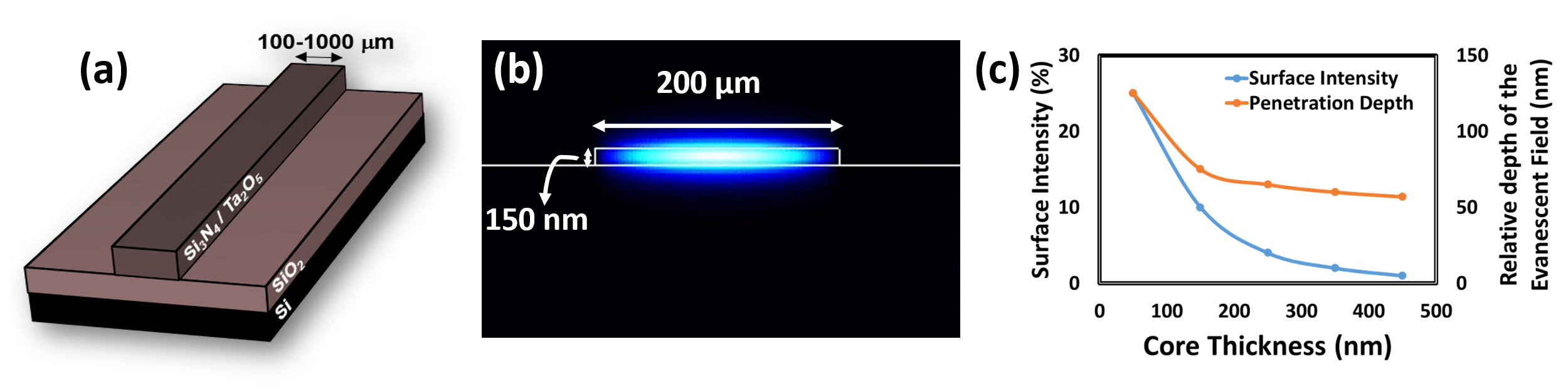


**Figure S1. Evanescent field simulations on a Ta_2_O_5_ strip waveguide.** **(a)** Schematic diagram of a photonic chip with a strip waveguide core width varying from 100 µm to 1000 µm. **(b)** Simulated field distribution of a fundamental transverse electrical (TE) mode on a Ta_2_O_5_ waveguide of 200 µm wide and 150 nm thickness. **(c)** The strength (surface intensity) and the penetration depth of the evanescent field vary as a function of the waveguide thickness. The wavelength considered in the simulation corresponds to 561 nm and the waveguide core material Ta_2_O_5_.

1. Sample preparation work-flow for chip-TIRFM of Tokuyasu sections

Sample preparation plays a key role in the imaging outcome of chip-based microscopy. Figure S2 provides a schematic workflow of the preparation steps for chip-TIRFM imaging of placental cryosections per the Tokuyasu method. The protocol is based on an existing procedure for fluorescent labeling of Tokuyasu sections on glass coverslip^53^. From the orange-dotted line in Figure S2a, all the steps are optimized according to the specific needs of each sample. In particular, the washing steps of the cryoprotectant (step after orange box in Figure S2a) can be carried out at temperatures ranging from 0 °C to 37 °C.


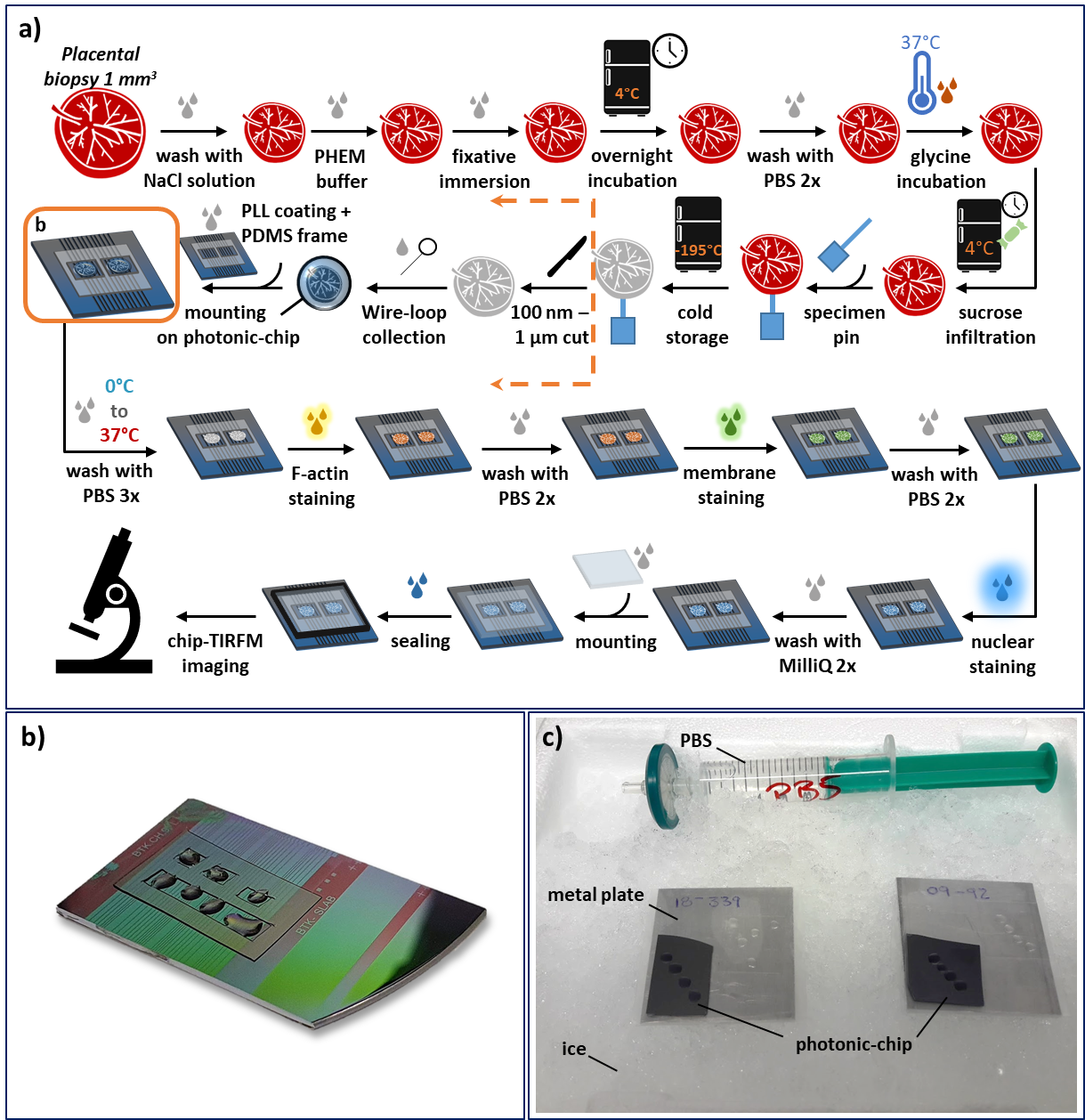


**Figure S2. Sample preparation protocol for fluorescence staining of Tokuyasu cryosections on a photonic chip.** **(a)** Schematic workflow of the sample preparation steps per the Tokuyasu method of a placental section. **(b)** Depiction of the orange box in (a) illustrating a photonic chip with Tokuyasu cryosections on top and surrounded by a custom-made PDMS frame. **(c)** The photonic chips are placed on top of metal plates on ice for the washing step in cold PBS following the orange box in (a).

1. Mode-averaging for homogeneous illumination in chip-TIRFM imaging

The waveguides used for tissue imaging are wide, supporting the propagation of several light modes (Figure S3a). Upon coupling of the excitation beam onto the waveguide, a non-uniform intensity distribution is observed due to multiple mode interference (MMI) patterns (Figure S3b). These patterns change depending on the position of the coupling objective. To achieve isotropic illumination of the specimen, the coupling objective is scanned along the input facet of the chip while individual frames are acquired (Figure S3c). The collected image stack is averaged (Figure S3d) and then deconvolved (Figure S3e) to obtain a diffraction-limited chip-TIRFM image.

Interestingly, on-chip MMI patterns assist the IFON methods, especially for dense samples such as tissues. The spatio-temporal fluctuations from the sample are a decreasing function of the spatial density of the labels. For a dense tissue sample, this results in a higher average signal at the cost of low variance in the fluorescence intensity over time. This makes super-resolution imaging of tissue samples using IFON methods difficult. By using non-uniform MMI patterns not all regions (fluorophores) are excited at the same time, which helps to alleviate the issues with dense labeling. Furthermore, as these MMI patterns are generated inside the photonic chip, they carry high spatial frequencies due to the high refractive index of the waveguide material.


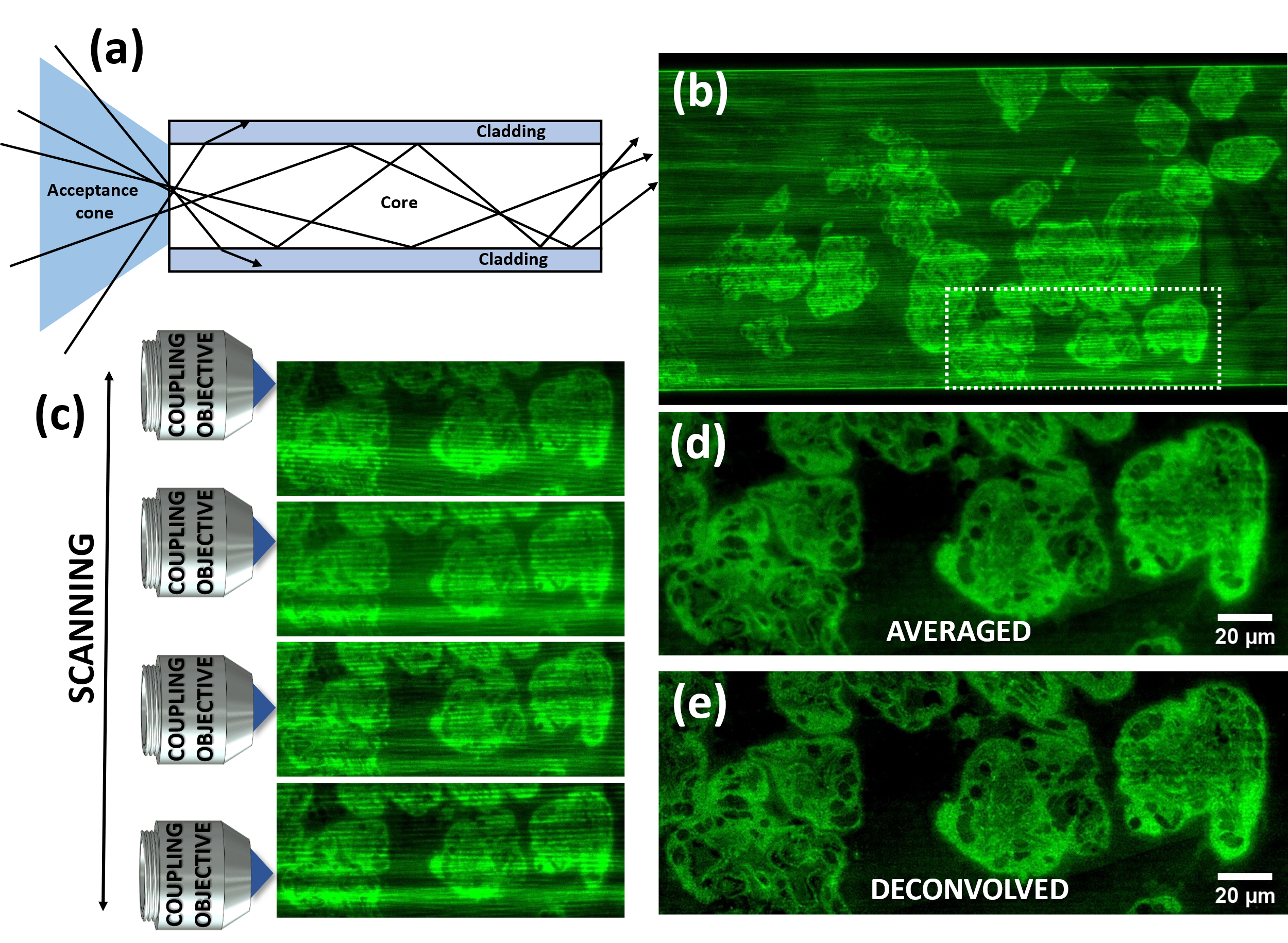


**Figure S3. Mode-averaging for chip-TIRFM imaging of a 400 nm thick human chorionic villi cryosection.** Membranes with CellMask Orange (pseudocolored in green). **(a)** Schematic diagram of a multi-mode waveguide supporting the propagation of multiple light modes. **(b)** Top view of a multi-mode pattern generated by the interference of multiple propagating light modes. **(c)** The excitation beam is scanned across the input facet of the waveguide while individual frames are collected (in this example four frames are illustrated). **(d)** The acquired stack is post-processed to obtain an averaged image with uniform intensity distribution. **(e)** The averaged image is deconvolved to obtain a high-contrast diffraction-limit chip-TIRFM image of the tissue section.

1. Photobleaching of membrane markers

To obtain an overall view of tissue sections, a membrane marker is desired. However, membrane probes exhibit a high affinity to the photonic chip surface, resulting in strong absorption of the propagating light along the waveguide. This phenomenon not only limits the excitation intensity reaching the sample but also introduces an undesired background signal that hampers the quality of the chip-TIRFM imaging. To overcome this problem, the power of the excitation beam is temporarily increased to photobleach the fluorescent molecules in the vicinity of the imaging waveguide. Although the emission intensity at the sample location is also reduced, the fluorescent signal of the non-bleached molecules deeper in the sample remains stable through the image acquisition, allowing continuous illumination over prolonged timescales (> 5 min). Arguably, this phenomenon is due to the decaying nature of the evanescent field (see Supplementary Information S1). We hypothesize that, since the illumination intensity of the evanescent field is significantly higher at the interface between the coupled waveguide and the sample, the fluorophores in its close vicinity are more susceptible to irreversible photodamage. Further away from the waveguide, the fluorescent markers are exposed to lower excitation intensities and, consequently, less prone to photobleaching. Depending on the coupling efficiency and the geometry of the waveguide, the bleaching process can take a few seconds (2 – 10 sec), to around 1 min. Figure S4 shows a 400 µm wide waveguide containing a placental tissue cryosection labeled with CellMask Deep Red. Consecutive frames (1-5) illustrate diverse time points of the bleaching process over a fixed field of view.


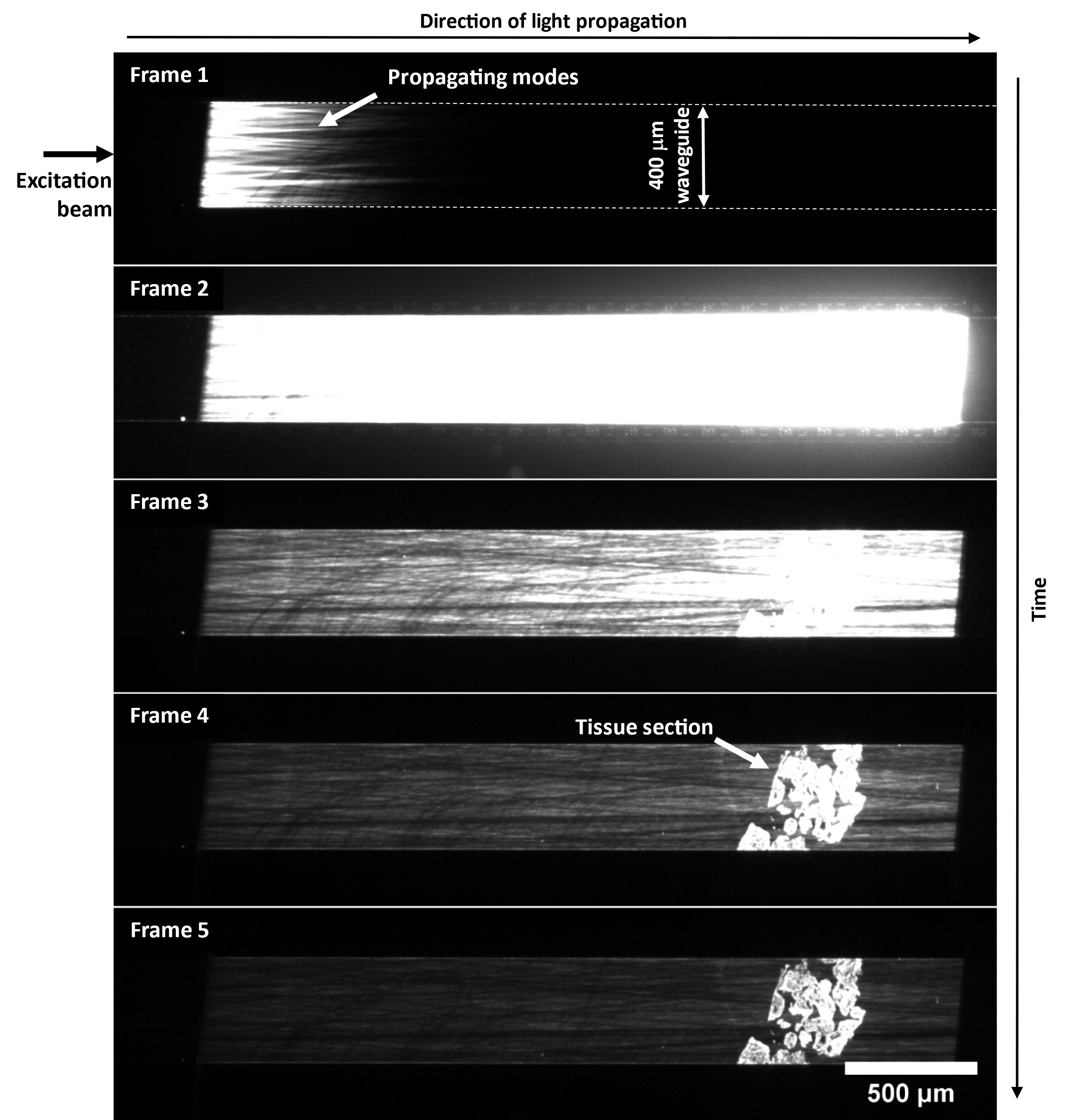


**Figure S4. Photobleaching process of a membrane marker bind to a 400 µm waveguide**. The power of the excitation beam is temporarily increased to induce photobleaching of the fluorescent molecules in the vicinity of the waveguide (frame 1 to frame 3). After a few seconds, the tissue section is revealed (frame 3 to frame 4). Further bleaching dramatically reduces the background signal of the membrane marker, allowing for high-contrast chip-TIRFM imaging (frame 5).

1. Sectioning artifacts

To conduct histological analysis, adequate morphological preservation is required. Sectioning artifacts in the form of knife marks (Figure S5a), tissue folds (Figure S5b), and tissue rupture (Figure S5c) are commonly present on the Tokuyasu cryosections. Sectioning parameters such as chamber temperature, slide thickness, and blade sharpness must be carefully adjusted to preserve the structure of the cryosections.


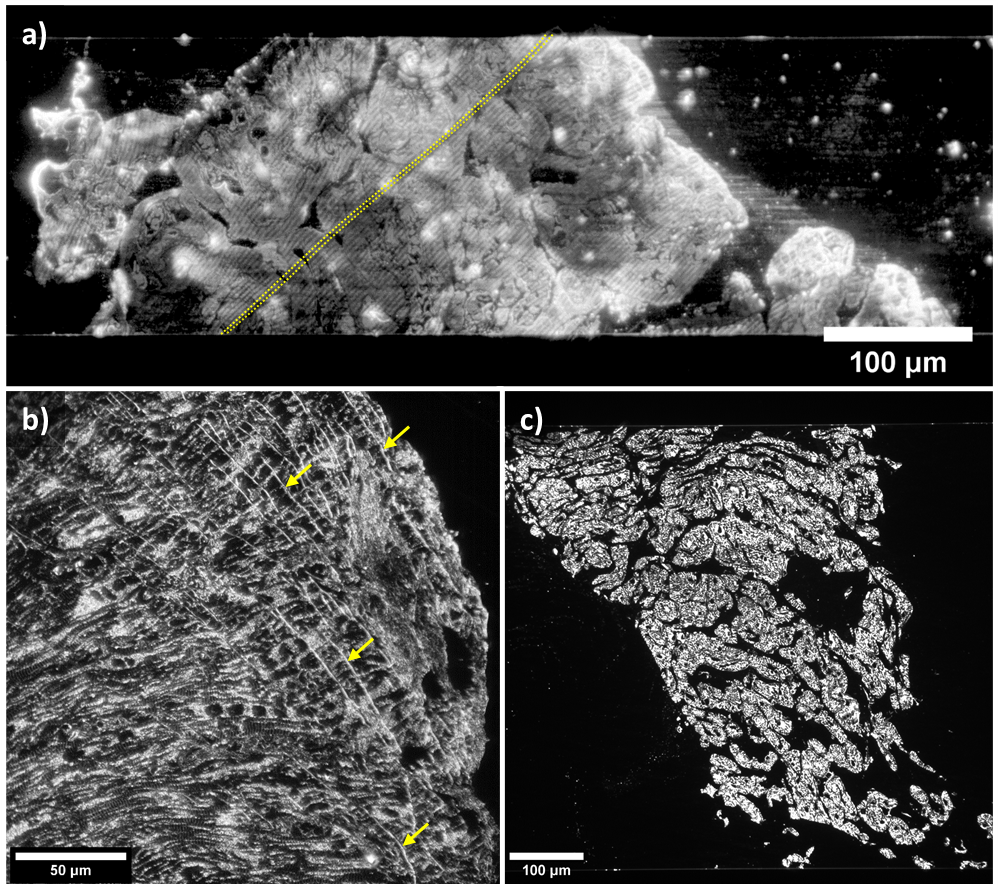


**Figure S5. Sectioning artifacts of Tokuyasu cryosections.** **(a)** The yellow-dotted lines illustrate the knife marks along a human placental cryosection. **(b)** The yellow arrows denote the location of folds on a pig heart cryosection. **(c)** Illustration of a pig heart tissue sample ruptured during cryosectioning.

1. Materials and reagents used for the preparation of Tokuyasu sections

Table S6a. Materials and reagents used for the preparation of Tokuyasu sections.

| Material/  reagent | Manufacturer | Catalog number | Stock concentration | Working concentration | Purpose |
| --- | --- | --- | --- | --- | --- |
| #1.5 coverslip | VWR | 48393-151 | - | - | Coverslip |
| Picodent twinsil | Picodent | 1300 1000 | - | 1:1 mixture of solution A and B | Dental cement. Gluing and sealing. |
| Poly-L-lysine | Sigma-Aldrich | P8920 | 0.1 % (w v^-1^) in H_2_O | 1:1 | Chip-surface coating for improved adhesion of biological samples |
| CellMask Deep Red (CMDR) | Invitrogen | C10046 | 5 mg mL^-1^ | 1:2000 | Membrane staining |
| Phalloidin-Atto565 | Sigma-Aldrich | 94072 | 27.88 mg mL^-1^ | 1:100 | F-actin staining |
| Sytox Green | Invitrogen | S7020 | 5 mM | 1:500 | Nuclear staining |
| Ethyl 3-aminobenzoate methanesulfonate  (Tricaine) | Sigma-Aldrich | E10521 | 98% | 1:1 | Euthanasia of zebrafish |
| rabbit anti-Tomm20 | Santa Cruz Biotechnology | FL-145 |  | 1:50 | Primary antibody for TOMM20 mitochondrial staining of the zebrafish eye retina |
| AlexaFluor 647 AffiniPure F(ab')2 fragment donkey anti rabbit IgG | Jackson Immuno-Research | 711-606-152 |  | 1:200 | Secondary antibody for TOMM20 mitochondrial staining in zebrafish eye retina |
| Texas Red-X Phalloidin | Invitrogen | T7471 |  | 1:50 | F-actin staining of zebrafish eye |
| Podoplanin (hamster anti-mouse) Monoclonal Antibody | ThermoFisher | 14-5381-85 | 0.5 mg mL^-1^ | 1:100 | Primary antibody for Podoplanin staining in mouse kidney |
| Goat anti-Hamster IgG (H+L) Alexa Fluor 568 | ThermoFisher | A-21112 | 2 mg mL^-1^ | 1:250 | Secondary antibody for Podoplanin staining in mouse kidney |
| Phosphate buffered saline (PBS) | Sigma-Aldrich | D8662 | - | 1:1 | Washing steps |
| Prolong Diamond | ThermoFisher | P36961 | - | 1:1 | Antifade mountant |

Table S6b. Buffers for sample preparation and imaging of Tokuyasu sections.

| Buffer name | Working concentration | Purpose |
| --- | --- | --- |
| PBG blocking buffer | 0.5% bovine serum albumin PBS (A6588.0050, Applichem) and 0.2 % gelatin type B (G-6650, Sigma) in PBS | Blocking buffer for immunolabeling of zebrafish eye for CLEM |
| Goat blocking buffer | 10% goat serum into 1% BSA in PBS | Blocking buffer for immunolabeling of mouse kidney for chip-TIRFM |
| SMLM imaging buffer | Oxygen scavenger system (0.5 mg mL^-1^ glucose oxidase (Sigma), 40 μg mL^-1^ catalase (Roche Applied Science), 10% (w v^-1^) glucose in phosphate buffer (pH 7.4), and 100 mM Mercaptoethylamine (MEA, Sigma) in PBS | Imaging buffer for SMLM experiments on mouse kidney tissue |

1. Chip-TIRFM imaging of immunolabeled mouse kidney samples

Fluorescent immunolabeling allows the identification of proteins of interest on the biological samples. The photonic chip not only withstands the chemical and thermal conditions of the sample preparation steps for Tokuyasu cryosections but also allows fluorescent immunolabeling of these samples. Figure S7 shows a 60X magnification image of a 400 nm thick Tokuyasu cryosection of a mouse kidney fluorescently labeled using CellMask Deep Red for membranes (shown in red) and Sytox Green for nuclei (shown in blue). The podoplanin protein was immunolabeled using hamster anti-mouse podoplanin as a primary antibody, and goat anti-hamster conjugated to Alexa Fluor 568 as a secondary antibody (shown in green). Supplementary Information S6 provides a detailed description of the dyes used for immunolabeling of the mouse kidney cryosection.

 
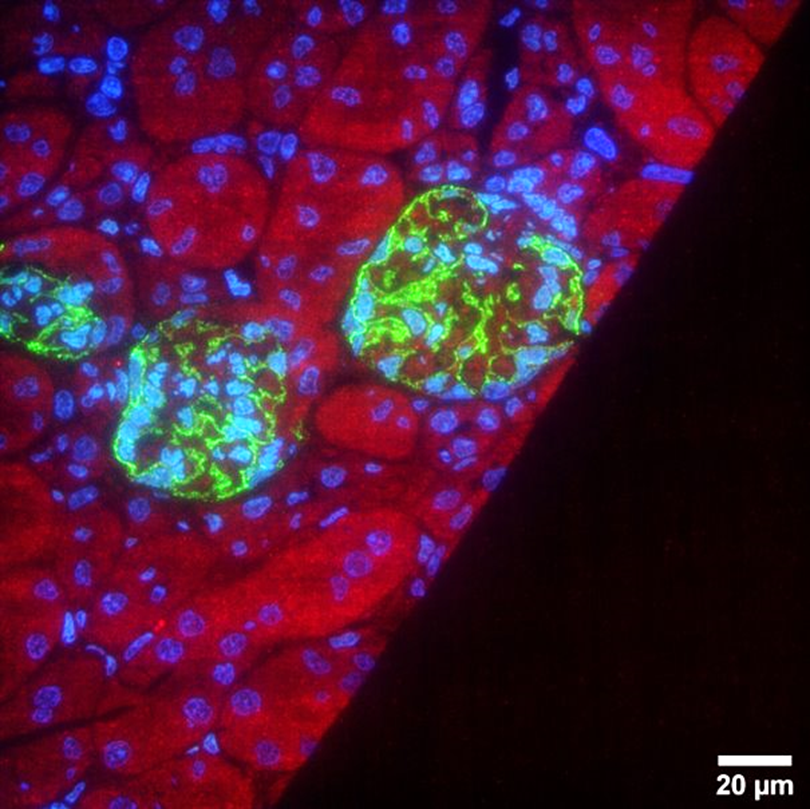


**Figure S7. Chip-TIRFM image of a 400 nm thick mouse kidney cryosection fluorescently immunolabeled by Tokuyasu method**. Membranes labeled with CellMask Deep Red (pseudo-colored in red) and nuclei labeled with Sytox Green (pseudo-colored in blue). The glomeruli were labeled with hamster anti-mouse podoplanin as a primary antibody, and goat anti-hamster conjugated to Alexa Fluor 568 as a secondary antibody (pseudo-colored in green). The image was collected with a 60X/1.2NA water immersion objective lens.

1. Chip-TIRFM imaging of immunolabeled human kidney samples

Figure S8 shows a chip-based multicolor TIRFM image of a 200 nm thick human kidney section prepared by Tokuyasu method. The sample was fluorescently labeled with Phalloidin ATTO565 for F-actin (displayed in magenta), and Sytox Green for nuclei (displayed in cyan). The mitochondria were immunolabeled using rabbit anti-TOMM20 mitochondrial marker as a primary antibody (Abcam, #AB186734), followed by donkey anti-rabbit AF647 as a secondary antibody (Invitrogen, #A31573). Figure S8c shows relevant kidney structures including proximal tubuli (PT), distal tubuli (DT), collecting duct (CD), microcapillary (white arrow), as well as the thin and the thick loops of Henle (white and yellow arrowheads, respectively). A magnified view of the white-dotted box in Figure S8c illustrates the improved contrast provided by MUSICAL (Figure S8e), as compared to the TIRF image (Figure S8d).


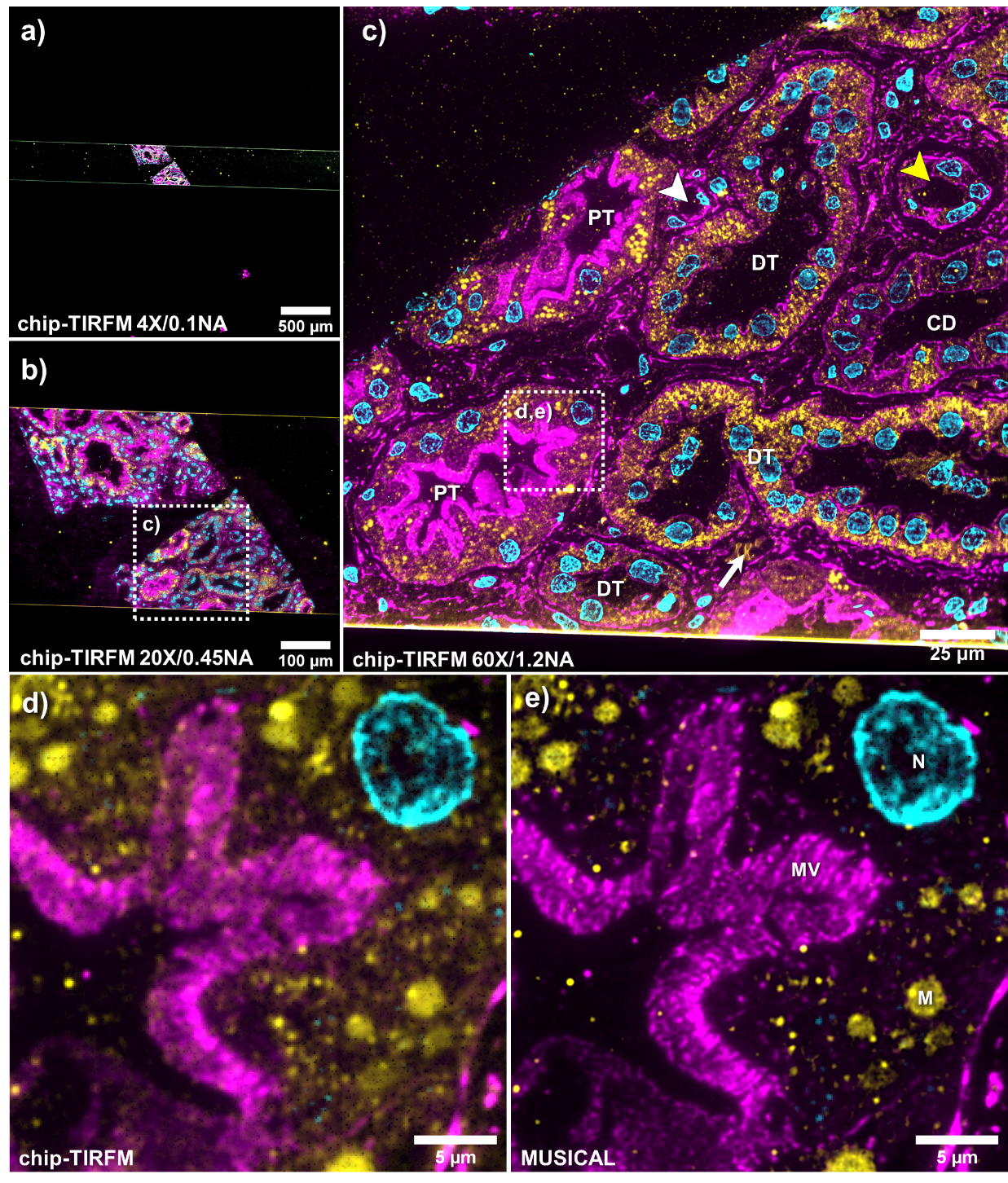


**Figure S8. Chip-TIRFM image of a 200 nm thick human kidney cryosection fluorescently immunolabeled by Tokuyasu method.** F-actin labeled with Phalloidin ATTO565 (pseudo-colored in magenta) and nuclei labeled with Sytox Green (pseudo-colored in cyan). The mitochondria were immunolabeled using rabbit anti-TOMM20 mitochondrial marker as a primary antibody, and donkey anti-rabbit conjugated to AF647 as a secondary antibody (pseudo-colored in yellow). **(a)** Large FOV chip-TIRFM image using a 4X/0.1NA microscope objective lens. **(b)** chip-TIRFM image using a 20X/0.45NA microscope objective lens. The white-dotted box illustrates the region of interest imaged in (c). **(c)** chip-TIRFM image using a 60X/1.2NA microscope objective lens showing proximal tubuli (PT), distal tubuli (DT), collecting duct (CD), microcapillary (white arrow), the thin loop of Henle (white arrowhead), and the thick loop of Henle (yellow arrowhead). The white-dotted box illustrates the region of interest magnified in (d,e). **(d)** Magnified view of the chip-TIRFM image in (c). **(e)** MUSICAL provides enhanced contrast over the chip-TIRFM image, allowing for clear visualization of the microvilli (MV), nucleus (N), and individual mitochondria (M).

1. Chip-TIRFM imaging of Pig heart tissue

The photonic chip allows for TIRF microscopy imaging of diverse cryo-preserved tissues from human and not human origin. Figure S9 shows a chip-based multicolor TIRFM of a longitudinal ultrathin (100 nm) tissue cryosection from a pig heart prepared by Tokuyasu method and fluorescently labeled with CellMask Orange for membranes (shown in magenta), and Sytox Green for nuclei (shown in cyan). The photonic chip allows high-contrast TIRF imaging with microscope objective lenses of low magnification and low numerical apertures (Figure S9a,b), a feature not available in conventional TIRF microscope objectives. A magnified view of the 60X/1.2NA objective (Figure S9d) reveals perinuclear mitochondrial clusters (white arrowhead), as well as mitochondrial rows (yellow arrowhead) aligned in parallel with the contractile units (the sarcomeres) of the tissue. Furthermore, since the CellMask Orange is a membrane stain, the transverse-tubules (t-tubules) also become visible in a characteristic periodic pattern, appearing as striations denoting the sarcomere length/spacing (white arrow in Figure S9d).


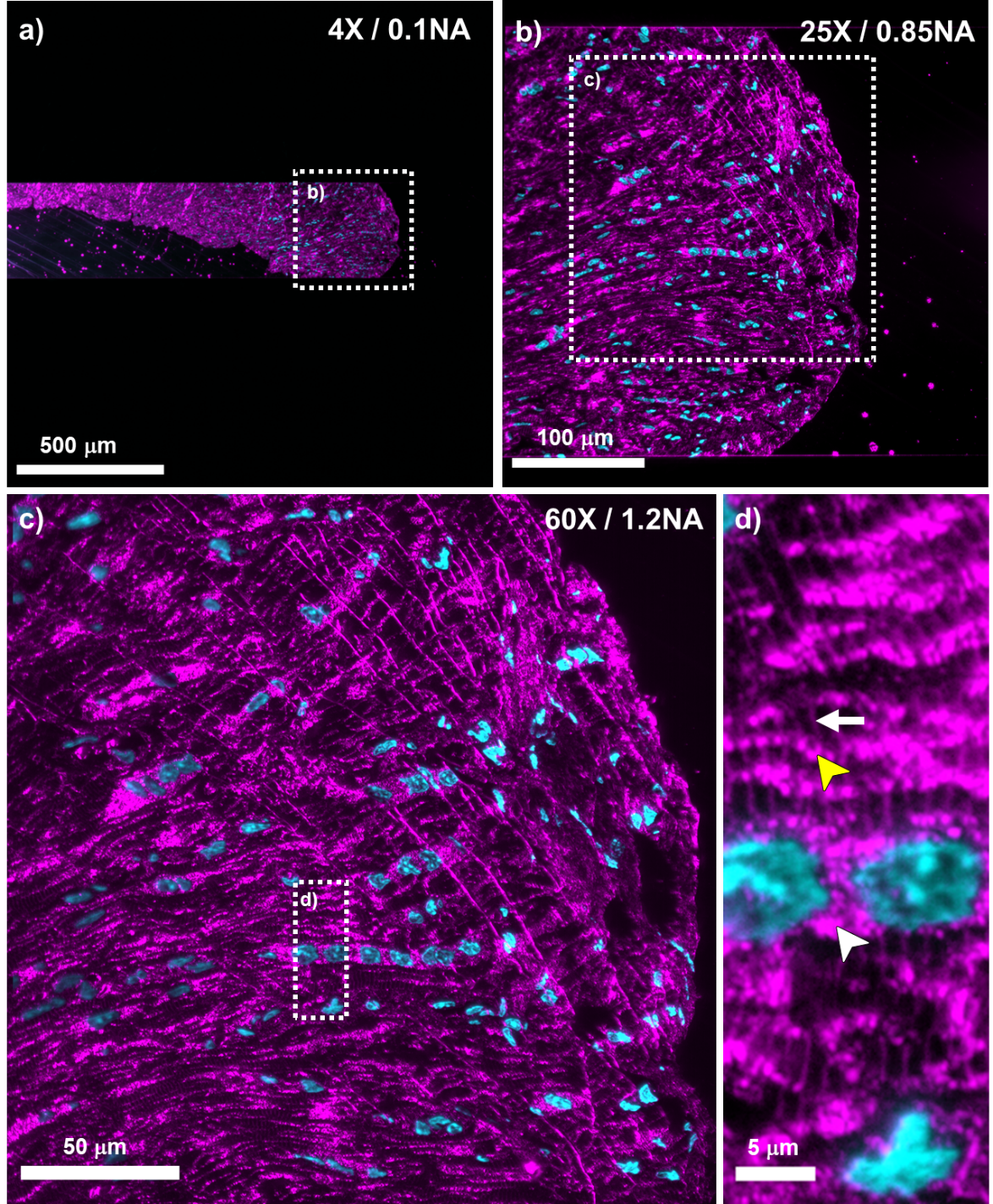


**Figure S9. Chip-TIRFM image of a 100 nm thick pig heart cryosection harvested from the left ventricle and prepared by Tokuyasu method.** Membranes labeled with CellMask Orange (pseudo-colored in magenta) and nuclei labeled with Sytox Green (pseudo-colored in cyan). **(a)** Large FOV chip-TIRFM image using a 4X/0.1NA microscope objective lens. The white-dotted box illustrates the region of interest imaged in (b). **(b)** chip-TIRFM image using a 25X/0.85NA microscope objective lens. The white-dotted box illustrates the region of interest imaged in (c). **(c)** chip-TIRFM image using a 60X/1.2NA microscope objective lens. The white-dotted box illustrates the region of interest magnified in (d). **(d)** A magnified view of the white-dotted box in (c) reveals prominent features of the cardiac tissue such as perinuclear mitochondrial clusters (white arrowhead), mitochondrial rows (yellow arrowhead) aligned in parallel with the sarcomeres, and the transverse-tubules (t-tubules) appearing as striations denoting the sarcomere length/spacing (white arrow).

1. Large FOV imaging of paraffin-embedded samples using chip-TIRFM

Formalin-fixed paraffin-embedding (FFPE) accounts for the most common preservation method employed for histological examinations worldwide. It entails chemical fixation of samples in formalin (e.g. an aqueous solution of formaldehyde) and subsequent embedding in paraffin to allow for long-time storage at room temperature. Preparation of FFPE samples for fluorescence microscopy imaging starts with sectioning the paraffin block into thin slices of approximately 4 µm in thickness. Thereafter, the sections are placed onto microscope glass slides for incubation in successive baths of xylene and rehydration in descent grades of ethanol. After staining with fluorescent probes, the sample is mounted with imaging media and covered with a glass coverslip before imaging under the microscope^53^. In chip-based microscopy, instead of a microscope glass slide, a photonic chip is used throughout the whole sample preparation and imaging process. Figure S10a shows a human placental FFPE sample sequentially imaged in TIRF mode over adjacent waveguides (denoted as “WG#”) using a 4X/0.1NA microscope objective. The individual images are merged into a large FOV TIRF image, as illustrated in Figure S10b. The dark horizontal stripes in Figure S10b correspond to the spacing between adjacent waveguides in the chip. The number of stripes can be minimized by: a) by reducing the spacing between waveguides to approx. 1 µm, and b) by increasing the waveguide width to approx. 3.5 mm (as a reference, the widest waveguide shown in Figure S10b corresponds to WG5, with a width of 1 mm). The acquisition process can be repeated over specific regions of interest with higher magnification/numerical aperture microscope objectives, to obtain sharper visualization of the tissue structure (Figure S10c,d). The dark patches observed in Figure S10c (shown with arrowheads) suggest sample detachment of the tissue section from the waveguide surface in these areas, impeding the evanescent field to reach and uniformly excite all the fluorescent molecules in the sample. Our future work will focus on optimizing the sample adhesion to improve chip-TIRFM imaging of FFPE sections.


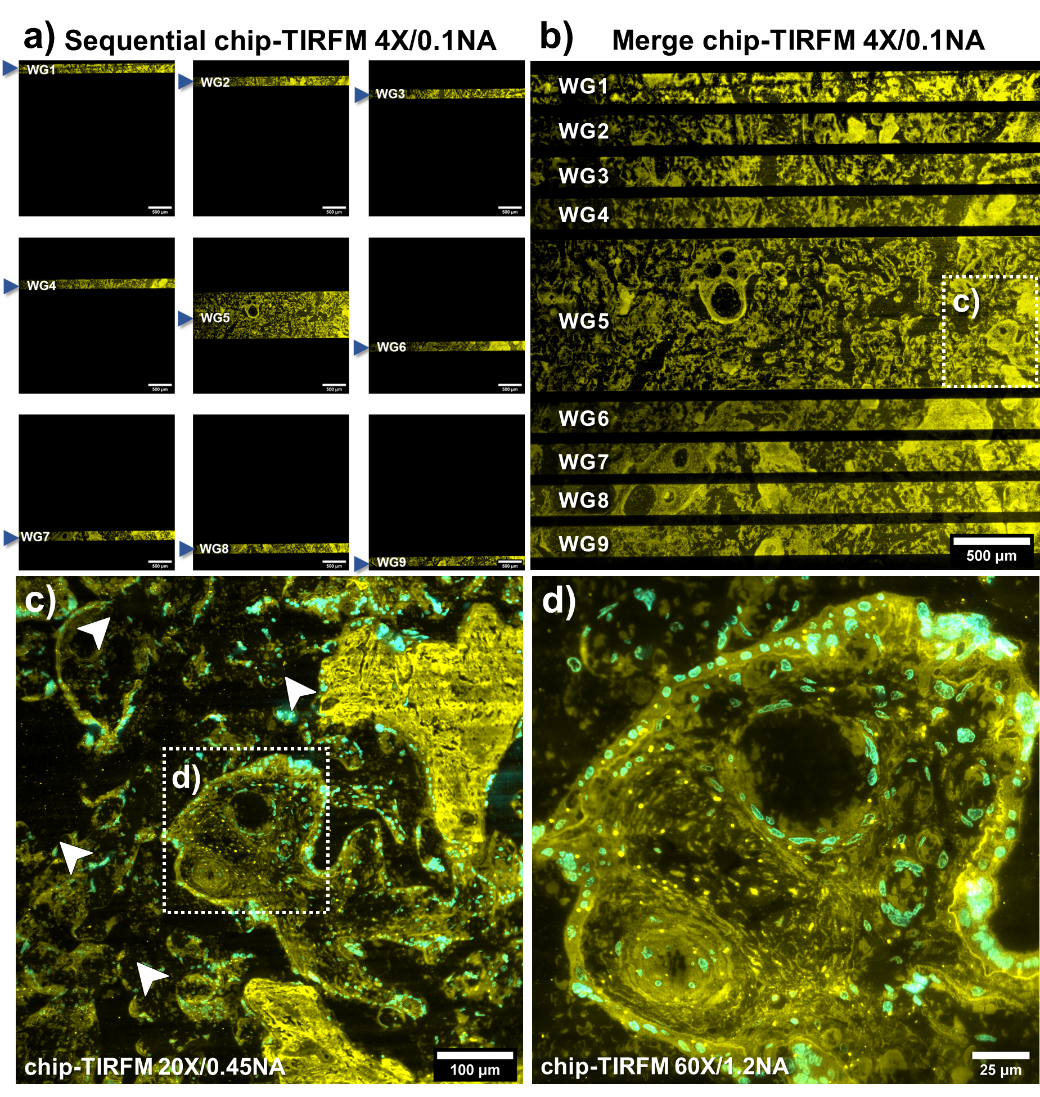


**Figure** **S10. Chip-TIRFM image of a 4µm thick FFPE human placenta**. Membranes labeled with MitoTracker Deep Red (pseudo-colored in yellow) and nuclei labeled with Sytox Green (pseudo-colored in cyan). **(a)** Sequential imaging in TIRF mode over adjacent waveguides (WG) using a 4X/0.1NA microscope objective. The blue arrowheads indicate the position of the coupling objective. **(b)** Large FOV merge of the TIRF images acquired in (a). The white-dotted box corresponds to the area imaged in (c). **(c-d)** TIRF images of the FFPE placental section using a 20X/0.45NA and a 60X/1.2NA microscope objective, respectively. The arrowheads in (c) indicate dark areas in the image as a consequence of sample detachment from the waveguide surface. Scalebar (a) 500 µm.

1. Quantification of resolution improvement based on decorrelation analysis

To quantify the resolution enhancement obtained with MUSICAL, we used decorrelation analysis^52^. This algorithm allows to estimate the resolution of single images based on the spatial frequency content and it is suitable for bandwidth-limited signals, as is the case of microscopy images. The decorrelation algorithm analyses the images under a series of high-pass filters that reduce the signal of interest to, eventually, leave it alone with the noise. As the noise is assumed to be in the entire spectrum, with this approach it is possible to estimate the point at which the signal is completely lost and, therefore, estimate the maximum spatial frequency at which it is still possible to obtain information from the sample. We used the MATLAB version of the algorithm to analyze both the average chip-TIRFM and the MUSICAL images, setting the number of sample points to 100 and the number of filters to 50. For the average chip-TIRF image, the estimated resolution was 268 nm, whereas for the MUSICAL image the resolution was estimated to be 195 nm. Importantly, the MUSICAL images were not post-processed to attenuate possible artifacts located in the high-end of the spatial frequency spectrum. Therefore, we expect the result to be an optimistic approximation of the resolution. Figure S11 shows the corresponding plots for resolution estimation per decorrelation analysis. The resolution is calculated with the formula $resolution={2\times pixel size}/{k_{c}}$, where $k_{c}$ corresponds to the maximum normalized spatial frequency shown in the plot (e.g. $k_{c}$ = 0.8053 for chip-TIRFM, and $k_{c}$ = 0.1111 for MUSICAL).


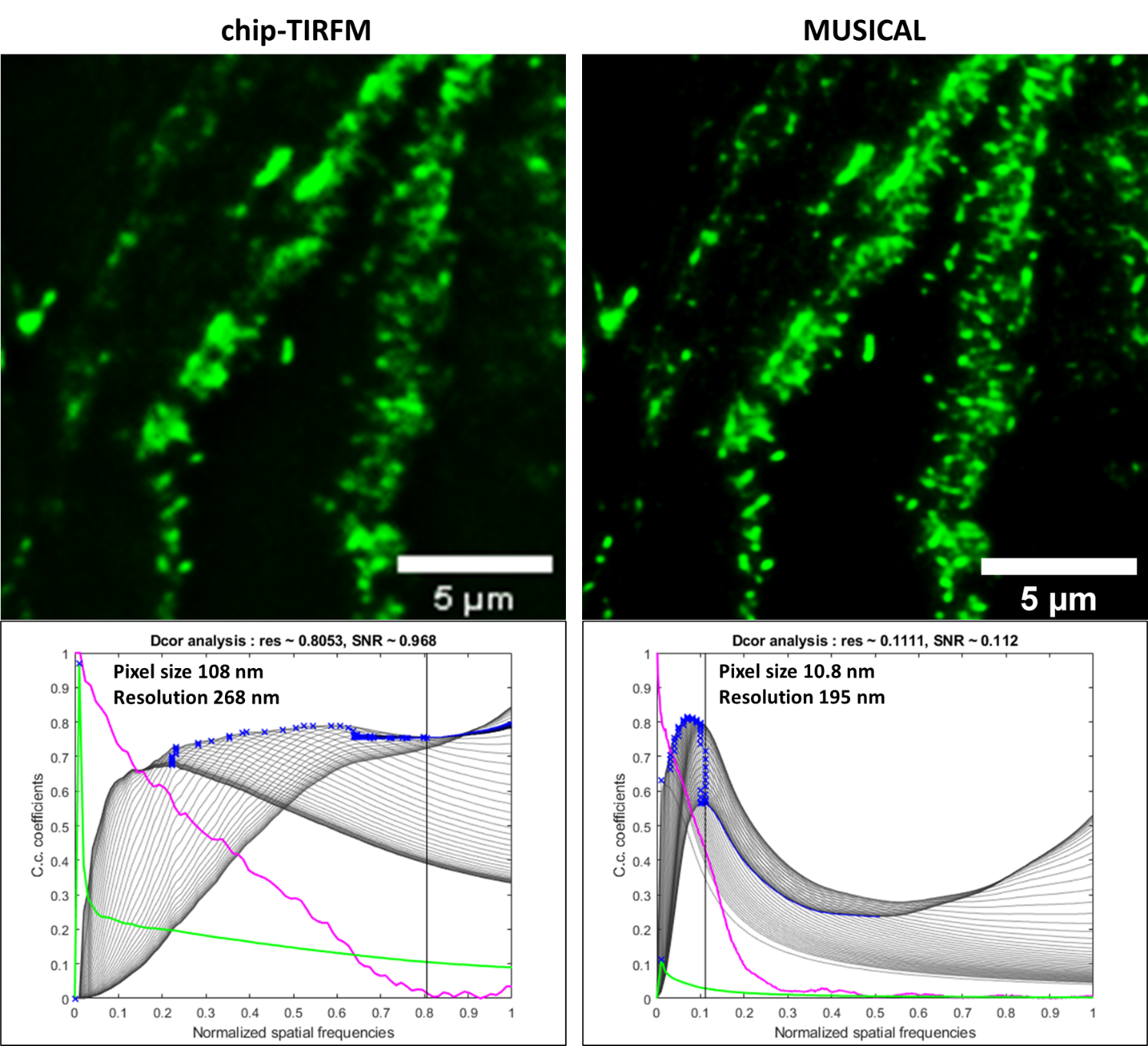


**Figure S11. Decorrelation analysis for resolution estimation of chip-TIRFM and MUSICAL images.** Placental chorionic villi tissue cryosection labeled with Phalloidin ATTO565 for F-actin (pseudocolored in green). The estimated resolution for chip-TIRFM is 268 nm, and 195 nm for MUSICAL. The MUSICAL image was adjusted with a logarithmic transformation in FIJI to improve its contrast.

1. Comparative FOV between chip-based IFON and SIM

The photonic chip allows the implementation of advanced microscopy techniques including intensity fluctuation-based optical nanoscopy (IFON) over large FOV (Figure S12a). Although structured illumination microscopy (SIM) has been proposed as the fastest super-resolution method for histopathological analyses^18,21,54^, the FOV achieved by this technique is limited to a much smaller area than chip-based IFON when a high magnification objective lens is used. A typical commercial SIM system, e.g., OMX V4 Blaze, GE Healthcare, allows for reconstructed 3D-SIM images of approximately 40 x 40 µm^2^ using a 60X/1.42NA oil immersion objective. Hence, to achieve a similar FOV to that of chip-based IFON, a tile mosaic composed of 7 x 7 reconstructed 3D-SIM images is needed (Figure S12b). Considering that a set of 15 raw images are required for each of the 8 z-planes necessaries to reconstruct a single SIM image, and accounting for the 10 µm overlap between adjacent images (Figure S12b), a total of 5880 SIM raw images are needed for an equivalent FOV as the one obtained with the photonic chip (15 raw images/z-plane x 8 z-planes/3D-SIM x 49 3D-SIM = 5880 raw images). Also, considering a typical image acquisition of 30 msec per raw image and a reconstruction time of 3 min per 3D-SIM image rounds up to a total imaging time of 2.5h from acquisition (30 msec/raw image x 5880 raw images = 176.4 sec $\approx$ 3 min) to 3D-SIM reconstruction (3 min/3D-SIM image x 49 3D-SIM images = 147 min). Importantly, we achieved a high-resolution chip-based IFON image over a fixed FOV of 220 x 220 µm^2^ after collecting a relatively small image stack of 500-frames using a 60X/1.2 water immersion objective, requiring approximately 10 min from acquisition to image reconstruction. We acknowledge that the implementation of a 2D-SIM scheme reduces the amount of acquired data (e.g., 9 raw images/2D-SIM x 49 2D-SIM = 441 raw images) and, consequently improves the processing time for a single-plane 2D-SIM, potentially becoming faster than chip-based IFON. We could not benchmark the exact numbers for this premise, since the SIM microscope available at our facilities only allows for 3D-SIM, and requires a z-stack of at least 7 to 8 planes to properly reconstruct an imaging area of 40 x 40 µm^2^. Nevertheless, the chip-based method offers a much less complex and highly cost-effective alternative to a commercial SIM instrument.


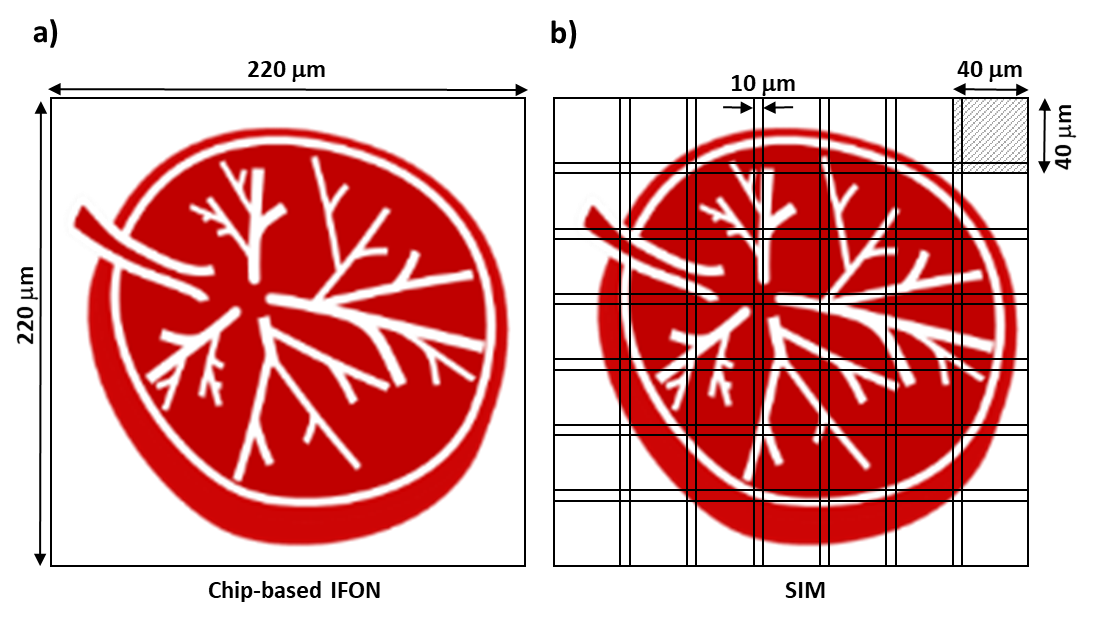


**Figure S12. Comparative FOV between chip-based IFON and 3D-SIM.** **(a)** Chip-based IFON allows a FOV of 220 x 220 µm^2^ after collection of an image stack of 500-frames and reconstruction time of approx. 10 min. **(b)** To achieve a similar FOV with 3D-SIM, a tile mosaic image is constructed. It requires the acquisition of 5880 raw images and a reconstruction time of 2.5h. The upper-right square denotes the typical FOV attainable with 3D-SIM (40 x 40 μm^2^).

1. Detailed description of the chip-TIRFM setup

The chip-TIRFM setup is composed of two main parts, namely the collection path and a photonic chip module, as illustrated in Figure S13a. The collection path consists of a commercial upright microscope equipped with an emission filter set (see Table S13), a sCMOS camera, and conventional microscope objective lenses of diverse magnifications, which can be interchanged depending on the imaging needs. Figures S13b and S13c provide a detailed view of the chip-TIRFM setup.

Table S13. Longpass and bandpass filters used in the setup for image acquisition

| Excitation wavelength (nm) | Emission filter set | |
| --- | --- | --- |
|  | Long-pass filter (nm) | Band-pass filter (nm) |
| 488 | 488 | 520/36 |
| 561 | 561 | 591/43 |
| 640 | 664 | 690/40 |


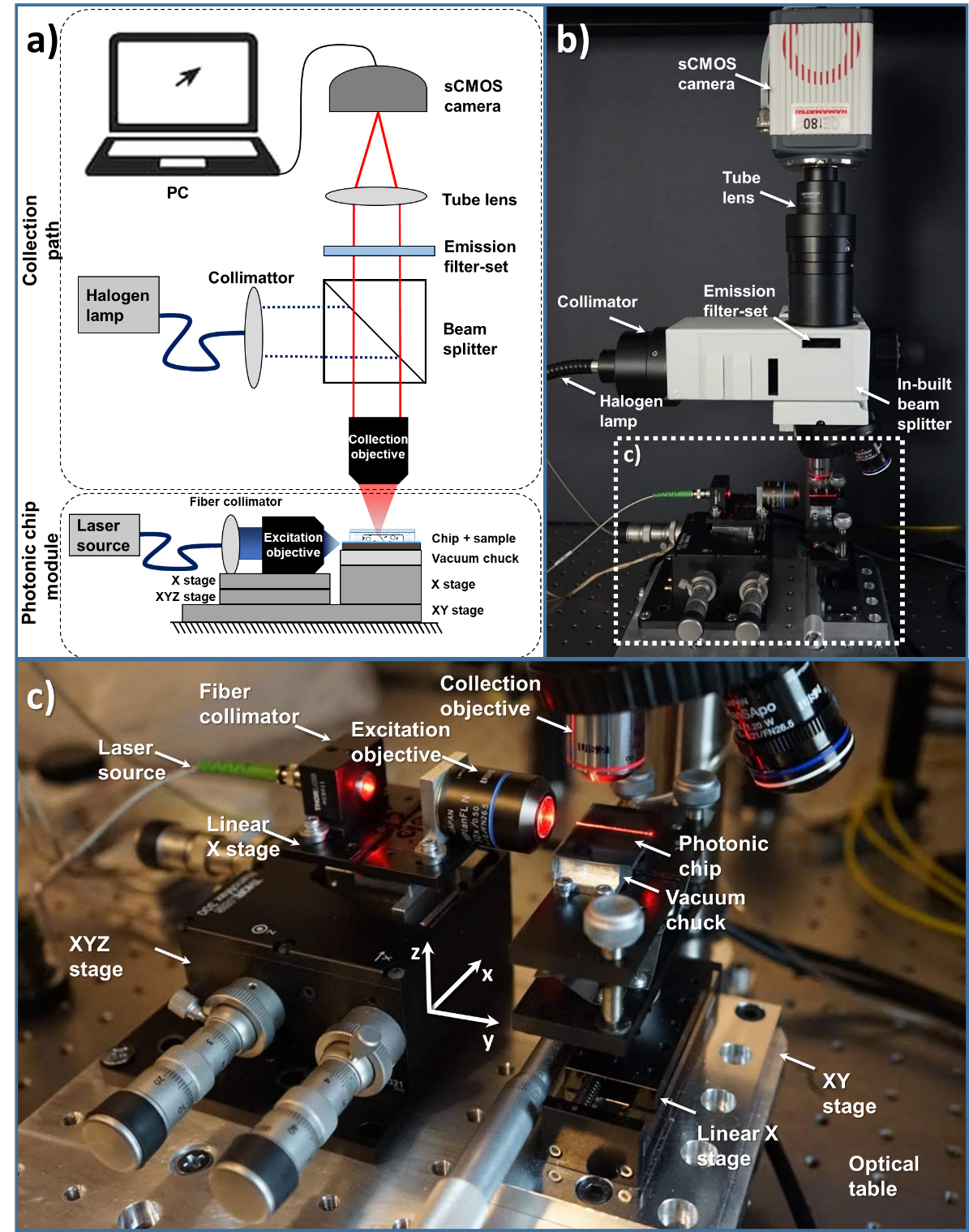


**Figure S13. Chip-TIRFM setup.** **(a)** Schematic representation of the chip-TIRFM setup illustrating the collection path and the photonic chip module. **(b)** Side view of a chip-TIRFM setup denoting the collection path components. The dotted-white box represents the photonic chip module shown in (c). **(c)** Close view of the photonic chip module components.

1. SEM imaging on a photonic chip

For correlative light-electron microscopy (CLEM), the Tokuyasu cryosections are imaged on a scanning electron microscope (SEM) after completion of chip-TIRFM imaging. To this, the coverslip and the PDMS frame are removed. Then, the sample is post-fixed with 0.1% glutaraldehyde, masked with methylcellulose, and further coated with a 10 nm layer of platinum/carbon. The photonic chip is placed on a 25 mm Pin Mount (Figure S14A) and transferred to a SEM. A bright-field image assists in finding the sample (Figure S14B). A low accelerating voltage allows high-resolution SEM imaging of the sample (Figure S14C).


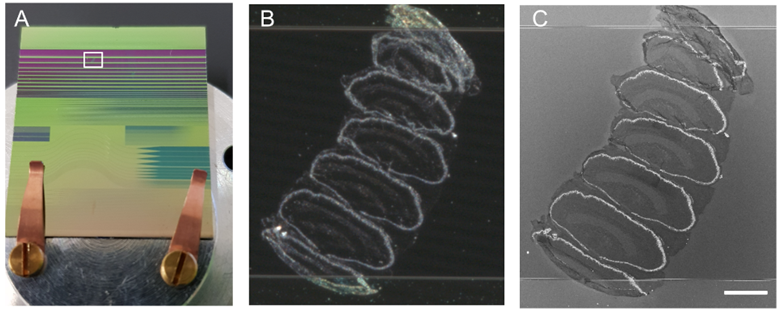


**Figure S14. Chip-based CLEM imaging.** **(A)** Photonic chip mounted on a 25 mm pin stub for imaging in the SEM. The white frame corresponds to the zoomed area in (B) and (C). **(B)** Bright-field image of zebrafish retina serial sections on a 600 µm strip waveguide. **(C)** The same area acquired with SEM. Scale bar 100 μm.

1. References – Supplementary Information

18. Wang, M. *et al.* High-resolution rapid diagnostic imaging of whole prostate biopsies using video-rate fluorescence structured illumination microscopy. *Cancer Research* **75**, 4032-4041 (2015).

21. Schlichenmeyer, T. C., Wang, M., Elfer, K. N. & Brown, J. Q. Video-rate structured illumination microscopy for high-throughput imaging of large tissue areas. *Biomedical Optics Express* **5**, 366-377 (2014).

52. Descloux, A., Grußmayer, K. S. & Radenovic, A. Parameter-free image resolution estimation based on decorrelation analysis. *Nature Methods* **16**, 918-924 (2019).

53. Villegas-Hernández, L. E. *et al.* Visualizing ultrastructural details of placental tissue with super-resolution structured illumination microscopy. *Placenta* **97**, 42-45 (2020).

54. Wang, M. *et al.* Gigapixel surface imaging of radical prostatectomy specimens for comprehensive detection of cancer-positive surgical margins using structured illumination microscopy. *Scientific Reports* **6**, 27419 (2016).
